# The impact of COVID-19 on health service utilization in sub-Saharan Africa—a scoping review

**DOI:** 10.1186/s44263-024-00083-0

**Published:** 2024-08-07

**Authors:** Elliot Koranteng Tannor, John Amuasi, Reinhard Busse, Daniel Opoku, Emmanuel Ofori, Kwadwo Faka Gyan, Minas Aikins, Kojo Hutton-Mensah, Priscilla Opare-Addo, Wilm Quentin

**Affiliations:** 1 Department of Global Health, School of Public Health, Kwame Nkrumah University of Science and Technology, Kumasi, Ghana; 2 Department of Medicine, School of Medicine and Dentistry, Kwame Nkrumah University of Science and Technology, Kumasi, Ghana; 3 German-West African Center for Global Health and Pandemic Preparedness (G-WAC), Kumasi, Ghana; 4 Global Health and Infectious Diseases Research Group, Kumasi Centre for Collaborative Research in Tropical Medicine, Kumasi, Ghana; 5 Department of Implementation Research, Global One Health Research Group, Bernhard Nocht Institute for Tropical Medicine, Hamburg, Germany; 6 Department of Medicine, Division for Tropical Medicine, University Medical Centre Hamburg-Eppendorf, Hamburg, Germany; 7 Department of Health Care Management, Technische Universität Berlin, Berlin, Germany; 8 Grey Nuns Community Hospital, Edmonton, Canada; 9 Department of Medicine, Komfo Anokye Teaching Hospital, Kumasi, Ghana; 10 Chair of Planetary & Public Health, University of Bayreuth, Bayreuth, Germany

**Keywords:** Sub-Saharan Africa, Health service utilization, COVID-19 pandemic

## Abstract

**Background:**

Despite comparatively low rates of COVID-19 admissions and recorded deaths in sub-Saharan Africa (SSA), the pandemic still had significant impact on health service utilization (HSU). The aim of this scoping review is to synthesize the available evidence of HSU in SSA during the pandemic, focusing on types of studies, changes in HSU compared with the pre-pandemic period, and changes among specific patient groups.

**Methods:**

The scoping review was guided by the methodological framework for conducting scoping reviews developed by Arksey and O’Malley. We identified relevant studies through a search of PubMed (MEDLINE), Embase, Scopus, and Web of Science. We then provided a general descriptive overview of the extracted data focusing on the types of studies, patient groups, and change in HSU.

**Results:**

We identified 262 studies reporting on HSU in 39 SSA countries. Studies were mainly quantitative (192; 73.3%), involving multiple centers (163; 62.2%), conducted in hospitals (205; 78.2%), and in urban settings (121; 46.2%). The median number of participants was 836.5 (IQR: 101.5–5897) involving 62.5% females. Most studies (92; 35.1%) focused on communicable diseases and mainly among outpatients (90; 34.2%). Maternal and child health studies formed the largest patient group (58; 22.1%) followed by people living with HIV (32; 12.2%). Change in HSU was reported in 249 (95.0%) studies with 221 (84.4%) studies reporting a decrease in HSU. The median decrease in HSU was 35.6% (IQR: 19.0–55.8) and median increase was 16.2% (IQR: 9.1–31.9). The patient group with the largest percentage decrease was cardiovascular diseases (68.0%; IQR: 16.7–71.1) and the lowest percentage decrease was in patients with infections (27.0%; IQR: 16.6–45.6).

**Conclusions:**

A large body of literature is available on the effects of the pandemic on HSU in SSA. Most studies report decreases in HSU during the pandemic. However, patterns differ widely across disease categories, patient groups, and during different time periods of the pandemic.

**Supplementary Information:**

The online version contains supplementary material available at 10.1186/s44263-024-00083-0.

## Background

The coronavirus disease 2019 (COVID-19) pandemic has had devastating effects on health systems globally and particularly in sub-Saharan Africa (SSA) [1, 2]. The pandemic further aggravated the pre-existing weaknesses of health systems in the region, which were already strained due to poor health infrastructure and low density of skilled workforce [3, 4]. Even though there have been comparatively low rates of COVID-19 hospitalizations and deaths reported in SSA [5], evidence points to the pandemic having significantly impacted health service utilization (HSU) in the region [6].

HSU has been defined as the process of seeking professional healthcare services, usually provided in the form of healthcare contacts, with the purpose of preventing or treating health problems [7]. Disruptions in HSU during the COVID-19 pandemic have been described at the global level, but the intensity of disruptions differed across countries depending on the level of income status (high-income versus low-income), the type of services provided (e.g., emergency care versus elective surgery), and the time period (2020 versus 2021) according to a World Health Organization (WHO) survey [8].

These disruptions also affected the management of chronic non-communicable diseases (NCDs) [9], which may have ultimately contributed to higher mortality in patients with NCDs who were infected with the severe acute respiratory syndrome coronavirus 2 (SARS-CoV-2) [10]. It is therefore essential to better understand the impact of COVID-19 on HSU in SSA and among specific patient groups.

The Andersen behavioral model [11] has been extensively used as a framework for the analysis of HSU which includes both individual and contextual factors. Individual factors can be classified into predisposing characteristics, enabling factors, and need factors [12, 13]. Predisposing characteristics include age, gender, marital status, and ethnicity. Enabling factors include educational status, income, employment status, household size, and health insurance. Need factors include disease severity, duration of illness, and the presence of acute illness. Contextual factors may have included prioritization of emergency services, introduction of COVID-19 services, and increase in staff workload [14].

Previous global reviews on changes in HSU during the pandemic have included very few studies from SSA [15–17]. However, several studies have become available relatively recently that report on changes in HSU during the pandemic in various sub-Saharan African countries [6, 14, 18–22]. Yet, a recent overview of the literature on the impact of the COVID-19 pandemic on HSU in SAA remains unavailable. We therefore aim to assess available evidence on HSU in SSA during the COVID-19 pandemic. More specifically, we focused on changes in HSU and changes in HSU among specific patient groups studied.

## Methods

We conducted this scoping review guided by the methodological framework by Arksey and O’Malley for conducting scoping reviews [23]. We followed the Preferred Reporting Items for Systematic Reviews and Meta-Analyses for Protocols extension for Scoping Reviews (PRISMA-ScR) checklist for reporting our findings [24, 25] (Additional file: Table S1). The study design and protocol has been published [26].

### Information sources and search strategy

We conducted a comprehensive search of all peer-reviewed literature published between December 2019 and March 2023. We identified relevant studies through a search of PubMed (MEDLINE), Embase, Scopus, and Web of Science. Our search strategy was built on the basis of synonyms related to three key concepts: (1) “COVID-19 pandemic,” (2) “health service utilization” and related synonyms, and (3) “sub-Saharan Africa” as the population of interest. We employed the Boolean operators “AND” or “OR” to combine and refine terms as appropriate. We used truncations and field tags to improve the efficiency of the search. Given the complex nature of HSU, we used synonyms that capture various health services such as prescription, surgeries, ante-natal clinic or care, dental services, clinic, admissions, consultations, emergency visits, hospital visits, nursing services, endoscopy, scan, and imaging. The Medical Subject Headings (MeSH) term for sub-Saharan Africa and the list of all the 46 countries in SSA according to the United Nations (UN) were included in the search [26] (Additional file 2: Table S2).

### Eligibility criteria

We included studies that reported on HSU in SSA during the COVID-19 pandemic. The period of the COVID-19 pandemic was operationally defined from 11th March 2020 (when the WHO declared the pandemic) [27] to 31st March 2023. Two reviewers among a set of reviewers (KHM, POA, KFG, EO, and MA) independently screened each article for potential inclusion into the study. A third reviewer (EKT) was consulted in cases of disagreement for resolution. The review process involved first the screening of the title and abstract and second a detailed full text review for eligibility.

Each full text review was also done by two independent reviewers (KHM, POA, KFG, EO, MA), and any conflicts were resolved by a third reviewer (EKT) for eligibility for extraction. All reviews and extractions were done with the Covidence software [28]. Articles selected meet the following criteria:

1. Types of publications: original research studies on health service utilization
2. Types of studies: single and multi-center studies, quantitative, qualitative, and mixed methods
3. Population of studies: patients, health care providers, and healthcare managers
4. Intervention: any reported intervention of health service utilization
5. Comparator: pre-pandemic health service utilization if reported
6. Outcome: health service utilization, change in health service utilization, and patient reported outcomes
7. Language: English and French
8. Data collection: primary and secondary data
9. Location of study: sub-Saharan African countries, hospital based, community based, or online studies
10. Time: studies published between 1st December 2019 and 31st March 2023

The following were excluded:

1. Guidelines, letters to the editor, research protocols, abstracts, recommendations, and reviews (systematic, scoping, and literature)
2. Multi-center studies with one country outside sub-Saharan Africa
3. Research protocol papers, pre-prints, or conference abstracts
4. Articles with no clear quantitative or qualitative data on health service utilization

### Data items and data extraction process

Results from the search were extracted from the Covidence software [28] and exported to Microsoft Excel for cleaning. We then followed the recommended data charting method proposed by Arksey and O’Malley [23] to extract the relevant details of included studies. Double extraction was used for a 10% sample of randomly selected studies for inclusion, and any conflicts were resolved by a third reviewer (EKT). Data was extracted under the following themes: (i) the characteristic of the study population, (ii) methods used for data collection, (iii) definition and measures of HSU and patient groups studied, and (iv) changes in HSU and reported changes in specific patient groups.

### Collating, summarizing, and reporting of the results

All the extracted data were reviewed to ensure completeness and accuracy before analysis. For the quantitative studies, the median and interquartile ranges of studies reporting increases or decreases in HSU were analyzed. Analyses were performed (1) for all patients, (2) for the specific patient groups studied, and (3) for those studies that reported on changes in HSU. Being a scoping review, data was not pooled for further systematic meta-analysis.

### Risk of bias assessment or quality appraisal

Risk of bias assessment or quality appraisal was not performed for the included studies following existing guidance for scoping reviews [23].

## Results

### Characteristics of included studies

**Fig. 1:**
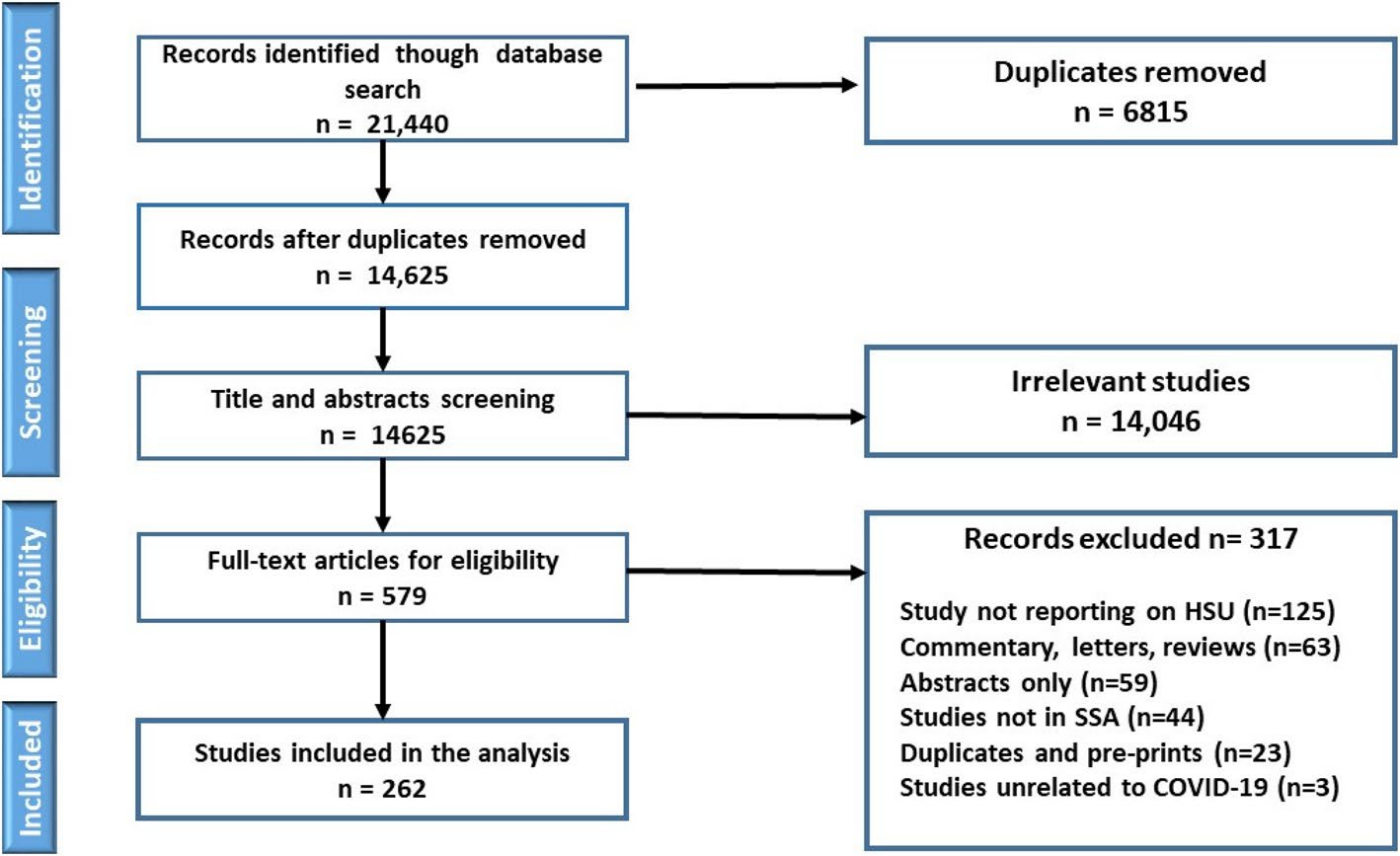
PRISMA study flow diagram

A total of 21,440 articles were identified from the databases and after removal of duplicates, 14,625 articles were available for title and abstract screening with 579 articles eligible for full text review. Data from 262 articles were extracted for analysis which were all in English (Fig. 1) (Additional file 3: Table S3).

Table 1 summarizes study characteristics of included studies. Slightly less than half of all studies were published in 2022 (116; 44.3%). Almost half of all included studies had a study duration of less than a year (126; 49.0%) and the median study duration was 364.5 (IQR 89–730) days. Almost three-quarters (192; 73.3%) were quantitative studies, while 39 (14.9%) were qualitative. Over a half of all the quantitative studies were retrospective (117; 60.9%), while almost a third (64, 32.8%) were described as observational studies. There were two quasi-experimental studies and one randomized controlled trial in the included studies.

**Table 1:** Characteristics of the included studies on health service utilization in sub-Saharan Africa (*n* = 262)

Study variable	Frequency	Percentage
**Year of publication (*n* = 262)**		
2020	34	13.0
2021	94	35.9
2022	116	44.3
2023 (January to March)	18	6.9
**Duration of study (*n* = 257)**		
Less than a year	126	49.0
Between 1 and 2 years	59	23.0
2 years and above	72	28.0
**Median duration of study (days; median [IQR]) (*n* = 257)**	364.5	(89–730)
**Study design (*n* = 262)**		
Quantitative	192	73.3
Qualitative	39	14.9
Mixed	31	11.8
**Type of quantitative studies (*n* = 192)**		
Retrospective	117	60.9
Observational	63	32.8
Cohort study	9	4.7
Quasi-experimental study	2	1.0
Randomized control trial	1	0.5
**Qualitative data collection (*n* = 70)**		
Focus group alone	3	4.3
Focus group and interview	12	17.1
Interviews alone	55	78.6
**Types of data collected (*n* = 262)**		
Primary data	101	38.6
Secondary data	134	51.2
Both	27	10.3
**Number of countries (*n* = 262)**		
Single country	245	93.5
Multi-country study	17	6.5
**Single or multi-center study (*n* = 262)**		
Single center	88	33.6
Multi-center	163	62.2
Online/community survey	11	4.2
**World Bank classification assigned to countries (*n* = 39)**		
Low-income countries	21	53.9
Lower middle-income countries	14	35.9
Upper middle-income countries	4	10.2
**Scope of study (*n* = 262)**		
Hospital based	205	78.2
Community based	28	9.7
National	20	7.6
Other (online survey and regional)	9	3.4
**Health care setting of study (*n* = 262)**		
Primary	45	17.2
Secondary	39	14.8
Tertiary	82	31.3
Primary, secondary, and tertiary	26	9.9
Primary and secondary or tertiary	15	5.7
Community based	22	8.4
Not reported	33	12.6
**Location of study**		
Urban	121	46.2
Rural	43	16.4
Rural and urban	27	10.3
Peri-urban	16	6.1
Urban and peri-urban	2	0.8
Urban, peri-urban, and rural	10	3.8
Not reported	43	16.4

*IQR* Interquartile range, *n*, number of participants

More than half of all studies (134; 51.2%) used secondary data from medical records, while more than one-third (101; 38.6%) used primary data collection. In the qualitative studies, individual interviews were used for data collection in more than three-quarters of all studies (55; 78.6%) and focus group discussions alone were used in 3 (4.3%) studies with the remainder of studies using both.

**Fig. 2:**
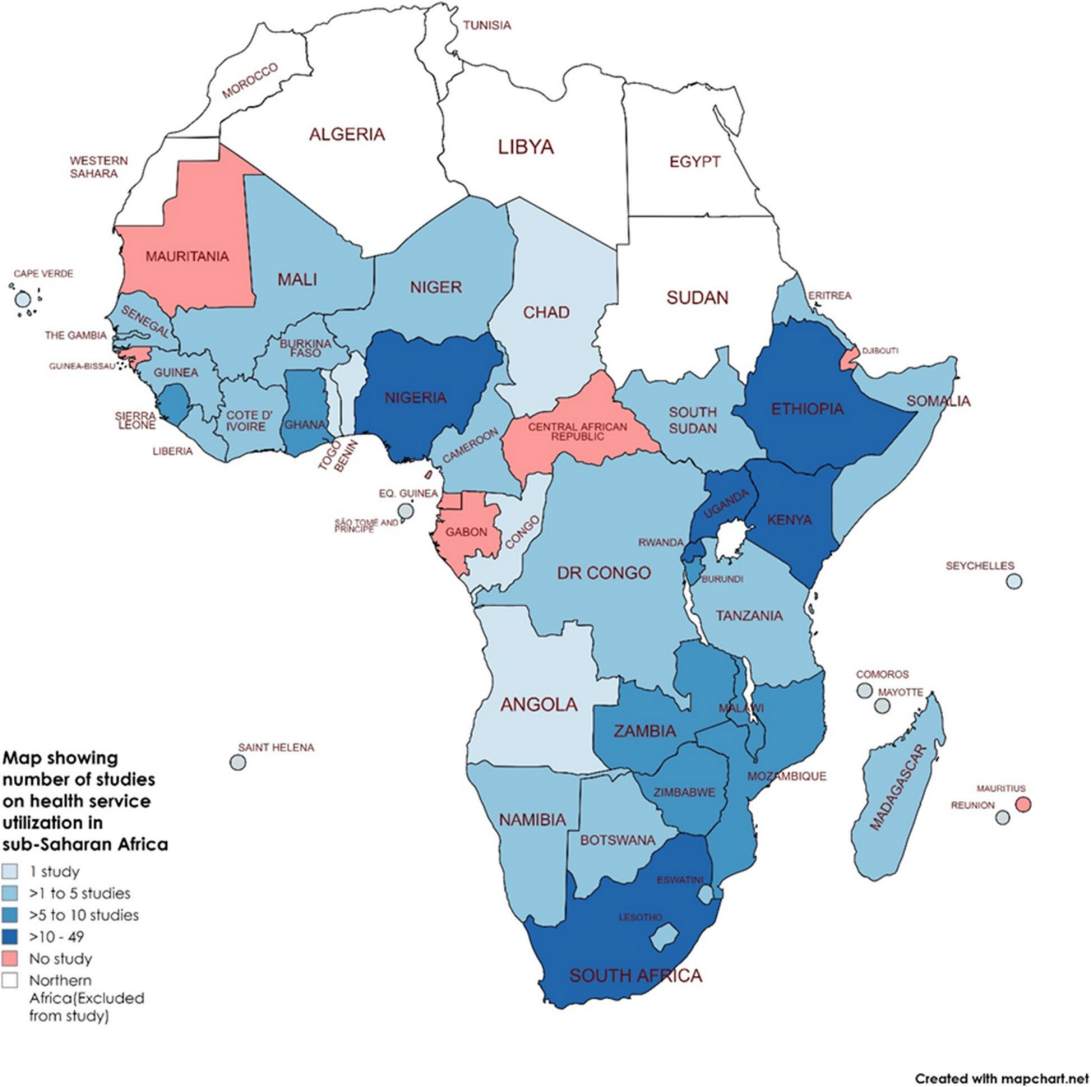
Number of studies on health service utilization during the COVID-19 pandemic in sub-Saharan Africa

Figure 2 provides an overview of the distribution of included studies across countries in SSA. Studies were conducted in 39 countries in SSA (84.8% of all SSA countries)—including 21 (53.9%) low-income countries, 14 (35.9%) lower middle-income countries, and 4 (10.3%) upper middle-income countries. Almost all studies (245; 93.5%) were conducted in one country and only 17 studies (6.5%) included two or more countries. Almost one-fifth of studies were conducted in South Africa (49; 18.7%) and a similar number of studies were conducted in Ethiopia (48; 18.3%) (Fig. 2).

More than three-quarters of all studies were hospital-based (205; 78.2%), with tertiary institutions accounting for almost one-third of all studies (82, 31.3%) (Table 1). Almost two-thirds of studies (163; 62.2%) were multi-center studies. Almost half of all studies were conducted exclusively in urban settings (121, 46.2%), while only 43 (16.4%) were conducted exclusively in rural areas.

Table 2 provides an overview of participants and disease groups studied by the included articles. The number of study participants ranged from 10 in a qualitative study to 99,600,000 in a national database. Age of included participants ranged from 4 days to 73.4 years in studies providing information on the age range (*n* = 94). Mean age of participants was 33.3 years (SD 12.7). Almost two-thirds of all participants (62.5%) were females in studies with the gender reported (*n* = 126). More than one-third of studies focused on communicable diseases (92; 35.1%) and another third focused on non-communicable diseases (89, 34.0%). The most studied patient groups were maternal and child health (58; 22.1%), followed by people living with HIV (32; 12.2%). Two-fifths of studies (105; 40.1%) were focused on curative care and one-fifth (54; 20.6%) on preventive health services (Table 2).

**Table 2:** Characteristics of participants and disease groups studies on health service utilization in sub-Saharan Africa

Variable	Frequency	Percentage
**Study participants (*n* = number of studies)**		
Number of study participants (*n* = 200) (range)	10–99,600,000	
Median number of participant *M* (IQR)	836.5 (101.5–5897)	
Age range of participants (years) (*n* = 94)	0.01–73.4	
Mean age of participants (years) *u* (± SD)	33.3 ± 12.7	
Proportion of female participants (% ± SD) (*n* = 126)	62.5 ± 26.1	
**Disease category studies (*n* = 262)**		
Communicable disease	92	35.1
Non-communicable disease	89	34.0
Both communicable and non-communicable diseases	53	20.2
Injuries	13	5.0
Preventive health	9	3.4
Other (blood donation, surgical, not disease)	6	2.3
**Participant/disease group studied (frequency is number of studies)**		
Maternal and child health	58	22.1
People living with human immunodeficiency virus (PLWHIV)	32	12.2
Healthcare managers and providers	31	11.8
Surgical	24	9.2
General inpatient and outpatient	18	6.9
Child health	16	6.1
Community	15	5.7
Tuberculosis	13	5.0
Cardiovascular disease, COPD, and chronic diseases	11	4.2
Sexual and reproductive health	10	3.8
Emergency and intensive care unit cases	8	3.1
Other infections (hepatitis B, C, malaria, urethritis)	7	2.7
Malignancies	7	2.7
Others (blood donors, epilepsy, dermatology, refugees, PWD)	12	4.6
**Category of health service rendered in the study (*n* = 262)**		
Preventive	54	20.6
Emergency	13	5.0
Diagnostic (e.g., radiology and laboratory)	7	2.7
Curative	105	40.1
Curative and preventive	47	17.9
Surgical	29	11.1
Rehabilitation	3	1.2
Telemedicine/telehealth	4	1.5
**Group of participants studied**		
Outpatients	90	34.2
Inpatients	37	13.5
Both inpatients and outpatients	76	27.1
Inpatient, outpatient, and other healthcare providers	5	1.9
Others (blood donors, care givers, community members, general public)	15	10.2
Healthcare providers and managers	33	12.4
Not stated	6	2.6
**Studies reporting change in health service utilization**		
Yes	249	95.0
No	13	5.0
**Decrease/increase in health service utilization reported (*n* = 249)**		
Increase	25	9.5
Decrease	221	84.4
No change	3	1.2
**Studies reporting change with quantitative data for analysis (*n* = 249)**	167	67.1

*SD* Standard deviation, *IQR* Interquartile range, *n* number *COPD* Chronic obstructive pulmonary disease, *PWD* People with disability

### Changes in health service utilization

There were 249 studies (95.0%) reporting change in HSU, either with quantitative data (167; 67.1%) (Additional file 4: Table S4) or qualitative data (82; 32.9%). Most studies (221; 84.4%) reported a reduction of HSU during the pandemic, but some studies (25; 9.5%) reported an increase or no change (3; 1.2%) (Table 2).

**Fig. 3:**
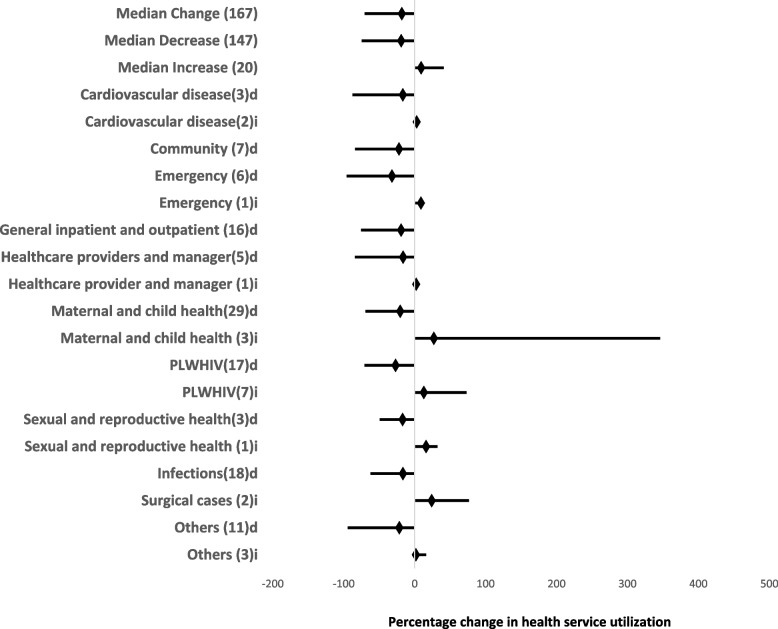
Forest plot showing changes in health service utilization as a result of the COVID-19 pandemic. PLWHIV, people living with human immunodeficiency virus; HSU, health service utilization; others include intensive care unit (ICU), blood donors, epilepsy, chronic obstructive pulmonary disease, elderly, dermatology, ophthalmology, people living with disability (PLWD), cancer, radiology, refugees; d, studies reporting decreases; i, studies reporting increases

Figure 3 provides more details on the 167 studies that reported a change with quantitative data available for analysis. The median percentage decrease in HSU reported in the 167 studies was 35.6% (IQR: 19.0–55.8). The largest number of studies was available for maternal and child health patients (29; 19.7%), followed by surgical patients (20; 13.6%), while relatively few studies were available for cardiovascular diseases (3; 2.0%) and sexual and reproductive health service utilization (3; 2.0%). The median reported percentage decrease was highest for cardiovascular conditions (68%, IQR: 16.7–71.1) and lowest for infections 27.0% (IQR: 16.6–45.6).

The overall median percentage increase in HSU was 16.2% (IQR: 9.1–31.9) reported in 20 studies. The largest group of studies reporting increases in HSU was among PLWHIV (people living with human immunodeficiency virus) with 7 studies (35% of studies reporting increases). The largest percentage increase in HSU was in surgical cases (38.3%, IQR: 24.0–52.5) with the lowest in studies involving health care providers and managers (2.2%) (Fig. 3).

## Discussion

This is the largest scoping review focusing on HSU during the COVID-19 pandemic in SSA to our knowledge. We found 262 articles reporting on studies conducted in 39 countries. The vast majority of included studies were quantitative (> 85%), with almost all studies reporting a change in HSU. The overall median reduction was 35.6%.

Our review shows that the impact of the pandemic on HSU in SSA has been extensively studied. This is surprising because a previously published systematic review [17] which reported studies up to August 2020 did not include any studies from SSA. Potential reasons for this may be because research in SSA emerged later than in other regions of the world or that studies were excluded because of perceived larger risk of bias.

With regard to the available studies, almost two-fifths were done in South Africa and Ethiopia, which may likely be due to the availability of established structures or expertise to support HSU research in these countries. However, no studies are available from seven countries in SSA, namely the Central African Republic, Djibouti, Equatorial Guinea, Gabon, Guinea-Bissau, Mauritania, and Mauritius. This means that there is no information yet on HSU during the pandemic in these countries in SSA.

Furthermore, our findings show that considerable knowledge gaps remain about the impact of the pandemic outside of hospitals and urban centers. Over three-quarters of included studies were hospital-based and about half were performed exclusively in urban centers. While the impact of COVID-19 was probably larger in urban areas because of higher population density and seeding effects [5], the relative lack of evidence available from primary health care levels and rural areas does not permit the full picture of the impact of the pandemic on HSU in Africa to be fully appreciated.

Our results are similar to findings from another systematic review on HSU including 81 studies from 20 high- and upper middle-income countries (no countries from SSA), which reported a 37% median reduction in overall HSU [17]. Thus, our findings corroborate the growing body of literature demonstrating that the impact of the pandemic on health systems in SSA was substantial. For example, a multi-center study in 63,954 facilities from eight countries in SSA (Cameroon, Democratic Republic of Congo, Liberia, Malawi, Mali, Nigeria, Sierra Leone, and Somalia) reported a decrease in maternal health services with significant declines in institutional deliveries, antenatal, and postnatal care [29]. Another multi-center study including 18 low- and lower middle-income countries estimated that reduction in HSU was associated with additional increase of 24.3% and 27.6% in maternal and child mortality respectively in the second quarter of 2020 compared to the pre-pandemic period [30]. These reductions in HSU were projected to be associated with excess mortality of 110,686 (3.6%) deaths in children under 5 years and excess maternal mortality of 3276 (1.5%) in the multi-center study [30].

We found large reductions in HSU for several groups of patients, such as cardiovascular diseases (68.0%), emergency services (48.5%), and child health (43.1%), and this may have contributed to increased morbidity and mortality during the pandemic. In particular, patients with cardiovascular diseases were more at risk of COVID-19 infection and had increased mortality [10]. In fact, the disruptions in HSU for NCDs may have contributed to the higher excess mortality during the pandemic in low-income settings (135 per 100,000) than in high-income settings (68.08 per 100,000) [31].

The reduction in HSU that was reported in most studies has been the result of disruptions in health service provision during the pandemic [6, 9]. According to Anderson’s behavioral model of HSU [11], individual and contextual factors can account for the reported changes in HSU [11, 32, 33]. Lockdown measures, lack of resources, shortage of personal protective equipment, fear of contagion, stigmatization, limitation of health service, reduction in effective health workforce due to COVID-19 infection, lower socio-economic status, and technological barriers are some reasons for reductions in HSU [15, 34–36].

Interestingly, several studies reported increases in HSU during the pandemic. The reasons for these increases are multifaceted, including catching up with backlogs in surgical care during less acute phases of the pandemic [37], deterioration of acute conditions (e.g., typhoid perforations as a result of delays in HSU during the peak of the pandemic) [38], or expanded access through introduction of updated guidelines for PLWHIV in later phases of the pandemic [39].

Our review has several limitations. First, we included studies from four major databases (PubMed (MEDLINE), Embase, Scopus, and Web of Science) but we may have missed studies and reports that were not published in peer-reviewed journals. Potentially, the inclusion of gray literature would have expanded the available evidence though authors were generally satisfied with the outcome after searching the major databases. Second, we did not perform a risk of bias assessment or appraise quality of included studies. However, this is in line with guidance for conducting scoping reviews [23, 40, 41], where the purpose is to provide an overview of the available literature rather than to summarize results of this literature. Our indicative findings about decreases in health service utilization should therefore not be mistaken as evidence on the size of the effect for different patient groups. Further systematic reviews and meta-analyses will be needed to specifically investigate the effects of the pandemic on HSU for particular groups of patients. Third, we did not consult any relevant bodies or stakeholders for the scoping review which could have potentially improved the study. Fourth, we categorized studies into groups based on broad classifications as stated by original authors, which may have reduced consistency of the classification.

## Conclusions

Our scoping review shows that a lot of research has been performed on HSU in SSA during the pandemic, but it also highlights several knowledge gaps, e.g., regarding certain countries, primary care levels, and rural areas. In addition, the impact of the pandemic seems to have been substantial for many groups of patients, as a lot of studies reported large decreases in HSU. The implications of these findings for researchers and policy-makers are that (1) efforts are needed to fill knowledge gaps about the effect of the pandemic in settings that have so far been underexplored, and this requires the establishment of structures and processes to ensure better data availability at the primary care level and in rural areas; (2) in order to safeguard service provision during future health crises, policy-makers should aim to strengthen resilience of health systems, addressing structural weaknesses, strengthening community-based service delivery models, and leveraging digital technologies; and (3) more research is needed to better understand the effects of the pandemic on HSU by (a) performing systematic reviews and meta-analyses of studies on particular groups of patients and (b) investigating the conditions that may enable health workers to provide health services during future health crises.

## Supplementary Information

Additional file 1. Table S1 Preferred Reporting Items for Systematic reviews and Meta-Analyses extension for Scoping Reviews (PRISMA-ScR) checklist

Additional file 2. Table S2 Key concepts, synonyms, and related terms to be used in the search strategy

Additional file 3. Table S3 List of included studies

Additional file 4. Table S4 List of studies included in the quantitative analysis

## Data Availability

All data generated or analyzed during this study are included in this published article and its supplementary files.

## Abbreviations

COVID-19: The coronavirus disease 2019
SSA: Sub-Saharan Africa
HSU: Health service utilization
HIV: Human immunodeficiency virus
NCDs: Non-communicable diseases
WHO: World Health Organization
SARS-CoV-2: Severe acute respiratory syndrome coronavirus 2
JBI: Joanna Briggs Institute
PRISMA-ScR: Preferred Reporting Items for Systematic Reviews and Meta-Analyses for Protocols extension for Scoping Reviews
MeSH: Medical Subject Headings
IQR: Interquartile range
SD: Standard deviation
PLWHIV: People living with human immunodeficiency virus
ICU: Intensive care unit
