# Supplementary material for: The impact of COVID-19 on health service utilization in sub-Saharan Africa—a scoping review
Source: BMC Glob Public Health. 2024 Aug 7;2:51. doi: 10.1186/s44263-024-00083-0 (PMC11622901; doi:10.1186/s44263-024-00083-0)
Supplement: Supplementary file 2 — Additional file 2. Table S2 Key concepts, synonyms, and related terms to be used in the search strategy [file 44263_2024_83_MOESM2_ESM.docx]

Table S2 - Key concepts, synonyms and related terms to be used in the search strategy

| What is the impact of Covid-19 pandemic on health services utilization in sub-Saharan Africa? | | | MESH terms |  | Search terms |
| --- | --- | --- | --- | --- | --- |
| Concept 1 | Covid-19 pandemic | #1 | MeSH “COVID-19”[Mesh] | OR | SARS-CoV-2 OR COVID-19 OR Coronavirus OR 2019-nCoV |
| AND | | | | | |
| Concept 2 | Health Services utilisation | #2 | MeSH “Facilities and service utilisation” [Mesh]   “Health Services”[Mesh]   “Patient Admission”[Mesh]   “Ambulatory Care”[Mesh]   “Immunization Programmes” [Mesh]   “Surgical Procedures, Operative”[Mesh]   “Telemedicine”[Mesh] | OR | “Facilities and service utilisation” OR “Health Services” OR “Patient Admission” OR “Ambulatory Care” OR surgery OR Telemedicine OR “Health service” OR “Health service utilization” or “Health service utilization” OR “Hospital care” OR Admission OR Out-patient OR outpatient OR outreach service* OR Vaccination* OR immunization OR Prescription OR medicine OR pharmac* OR antenatal OR postnatal OR “family planning” OR “Dental service” OR Nursing OR “nursing care” OR Telemedicine OR Telehealth OR Clinic OR Emergenc* OR Hospital OR Hospitalization OR hospitalization OR Endoscop* OR Scan OR Imaging OR Laboratory |
| AND | | | | | |
| Concept 3 | Sub-Saharan Africa | #3 | “Africa South of the Sahara”[Mesh] | OR | “sub-Saharan Africa” OR Angola OR Burundi OR “Central African Republic” OR Chad OR “Democratic Republic of Congo” OR Congo OR Rwanda OR Comoros OR Eritrea OR Ethiopia OR Kenya OR Madagascar OR Mauritius OR Seychelles OR Somalia OR Sudan OR Tanzania OR Uganda OR Botswana OR Eswatini OR Lesotho OR Malawi OR Mozambique OR Namibia OR “South Africa” OR Zambia OR Zimbabwe OR Benin OR “Burkina Faso” OR Cabo Verde OR Cameroon OR “Cote d Ivoire” OR “Ivory Coast” OR Equatorial Guinea OR Gabon OR Gambia OR Ghana OR Guinea OR Guinea-Bissau OR Liberia OR Mali OR Mauritania OR Niger OR Nigeria OR “Sao Tome and Principe” OR Senegal OR “Sierra Leone” OR Togo |
|  | Final strategy | #4 | #1 AND #2 AND #3 |  |  |

- MeSH, Medical Subject Headings.
